# Jiawei Qi Gong Wan Improves Endometrial Receptivity in PCOS-like Mice by Attenuating Inflammation: A Multi-Omics Study Linking Gut Microbiota and Metabolite Profiles

**DOI:** 10.3390/ph19081153

**Published:** 2026-07-24

**Authors:** Ruqun Zheng, Jinlong Song, Jie Li, Yingyan Shen, Qiqi Liu, Mengjia Shi, Yuxuan Zhuo, Haoyu Luo, Jing Li, Hongxia Ma, Min Hu, Chi Chiu Wang, Juan Li

**Affiliations:** 1 The Key Laboratory of Advanced Interdisciplinary Studies Center, Department of Traditional Chinese Medicine, The First Affiliated Hospital of Guangzhou Medical University, Guangzhou 510120, China; ruqunZheng@link.cuhk.edu.hk (R.Z.); 18718547987@163.com (J.L.); syenyingyan@163.com (Y.S.); liuqiqi15023921027@163.com (Q.L.); mengjia_shi2024@163.com (M.S.); 13502253900@163.com (Y.Z.); haoyu_luo0729@163.com (H.L.); doctorhongxia@126.com (H.M.); kindhumin@163.com (M.H.); 2 Department of Obstetrics and Gynaecology, The Chinese University of Hong Kong, Hong Kong, China; 3 The Key Laboratory of Advanced Interdisciplinary Studies, Department of Laboratory Medicine, The First Affiliated Hospital of Guangzhou Medical University, Guangzhou 510120, China; chasesjlo721@163.com; 4 State Key Laboratory of Respiratory Disease, National Clinical Research Center of Respiratory Disease, Guangzhou Institute of Respiratory Health, The First Affiliated Hospital of Guangzhou Medical University, Guangzhou Medical University, Guangzhou 510120, China; lijinghenan@163.com; 5 Li Ka Shing Institute of Health Sciences, School of Biomedical Sciences, Faculty of Medicine, The Chinese University of Hong Kong, Hong Kong, China; 6 The Chinese University of Hong Kong-Sichuan University Joint Laboratory for Reproductive Medicine, The Chinese University of Hong Kong, Hong Kong, China

**Keywords:** inflammation, metabolite, endometrial receptivity, Akt2/NF-κB

## Abstract

**Background/Objectives**: Uterine dysfunction contributes to infertility in PCOS. Jiawei Qi Gong Wan (JQGW) is used to improve endometrial homeostasis, but its mechanism is unclear. This study investigated whether JQGW improves uterine function via gut microbiota and metabolites. **Methods**: Letrozole-induced PCOS mice received JQGW (low/high dose), metformin, or vehicle for 35 days. Endometrial morphology, receptivity genes, Akt2/NF-κB signaling, gut microbiota (16S rRNA), and serum metabolites (LC-MS) were assessed. **Results**: PCOS mice showed reduced endometrial thickness (126.4 ± 10.8 μm vs. 215.6 ± 12.3 μm in controls, *p* < 0.001) and fewer glands (12.6 ± 1.8 vs. 28.4 ± 2.1, *p* < 0.001). JQGW-H increased endometrial thickness (189.3 ± 11.2 μm, *p* < 0.01 vs. PCOS) and gland number (23.1 ± 1.9, *p* < 0.01 vs. PCOS), restored the receptivity markers (*Nr2f2*, *Pc6*, *Ptch*, and *Hbegf*) toward normal levels, suppressed Akt2/NF-κB activation, and reduced inflammatory cytokines. JQGW shifted the β-diversity structure of the gut microbiota toward the control pattern, with *Oscillospira* enrichment (LDA > 4). Four metabolites (PA(20:0/16:1(9Z)), 7-methylguanosine, methoxyacetic acid, 8.11-eicosadiynoic acid) showed nominal elevation in PCOS and negative correlations with endometrial thickness (r = −0.73 to −0.89, unadjusted *p* < 0.01), although none survived FDR correction. **Conclusions**: JQGW ameliorates PCOS-associated uterine dysfunction, potentially via gut microbiota and metabolite modulation. Future studies should validate causality using fertility-based outcomes and microbiota transplantation.

## 1. Introduction

Globally, approximately 11–13% of women of reproductive age are affected by PCOS, a multifaceted endocrine disorder, with increased risks for metabolic, reproductive, and psychological comorbidities [1]. The pathogenesis of PCOS involves multiple factors, including elevated androgen levels, insulin resistance, and persistent low-grade inflammation [2]. PCOS is a major cause of infertility, with more than 75% of patients experiencing reproductive challenges [3]. Although the impact of PCOS on fertility is well documented, particularly regarding ovarian dysfunction and hormonal imbalances, its uterine aspects have only recently gained attention [4]. This clinical focus is warranted because women with PCOS continue to experience lower live birth rates despite the transfer of morphologically optimal blastocysts [5], suggesting that endometrial dysfunction is a key contributor to reproductive failure in this population [6,7].

The gut-uterus axis may have a significant impact on fertility, with evidence showing that maternal microbiota-derived metabolites affect embryo implantation [8,9]. Also, the gut microbiome contributes to nutrient processing and metabolite production [10], with these metabolites subsequently exerting effects on the host [11]. For example, prebiotic intake encourages the proliferation of beneficial gut bacteria, which in turn generate short-chain fatty acids (SCFAs), thus enhancing insulin sensitivity and reducing inflammation [12]. PCOS patients and animal models of PCOS have shown consistent signs of dysbiosis, characterized by altered microbial community structure and heightened inflammatory responses [13,14].

Currently, there are no established interventions for improving endometrial receptivity in women with PCOS [15], but by altering the gut microbiota and host metabolism, Chinese herbal medicine might be a viable treatment approach for PCOS [16]. In Traditional Chinese Medicine (TCM), Qi Gong Wan (QGW) is a classical herbal formula originally recorded in Yifang Jijie (Collected Explanations of Medical Prescriptions), a Qing dynasty medical compendium compiled by Wang Ang. QGW, a classical TCM formula indicated for the “phlegm-dampness in the uterus” pattern, is thought to improve endometrial receptivity by modulating a pathological microenvironment that corresponds to modern conditions such as PCOS with insulin resistance [17]. QGW includes eight herbs: Banxia (*Pinellia peltata* C.Pei), Chenpi (*Citrus reticulata* Blanco), Fuling (*Wolfiporia extensa* (Peck) Ginns), Chuanxiong (*Ligusticum chuanxiong* S.H.Qiu, Y.Q.Zeng, K.Y.Pan, Y.C.Tang & J.M.Xu), Xiangfu (*Cyperus rotundus* L.), Baizhu (*Atractylodes carlinoides* (Hand.-Mazz.) Kitam.), Shenqu (Massa Medicata Fermentata), and Gancao (*Glycyrrhiza uralensis* Fisch. ex DC.). Jiawei Qi Gong Wan (JQGW) is a modified formulation developed by incorporating Cangzhu (*Atractylodes lancea* (Thunb.) DC.), an herb employed in over 50% of PCOS prescriptions [18], to enhance the dampness- and phlegm-resolving capacity of the original QGW formula. Clinical studies have shown that QGW can improve metabolic dysfunction [19] in women with PCOS, and JQGW has been shown to reverse hepatic metabolic dysfunction in PCOS mouse models through the YAP/TAZ and Akt2-FoxO1 signaling pathways [20].

Although the metabolic effects of JQGW in PCOS are well established, whether and how this formula improves uterine function remains unknown, particularly regarding the role of the gut microbiota and its metabolites. To date, no study has investigated the potential link among JQGW, gut microbiota, and endometrial function in PCOS. To address this gap, we used a letrozole-induced PCOS-like mouse model and integrated endometrial functional assessments with 16S rRNA sequencing and untargeted metabolomics. The primary goal of this study was to determine whether JQGW improves uterine dysfunction in PCOS and, if so, to explore whether this effect is mediated through modulation of the gut microbiota and serum metabolite profile. Elucidating this gut–uterus axis may provide a novel mechanistic basis for JQGW as a therapeutic option for PCOS-related infertility.

## 2. Results

### 2.1. Establishment of the PCOS-like Mouse Model

**Figure 1:**
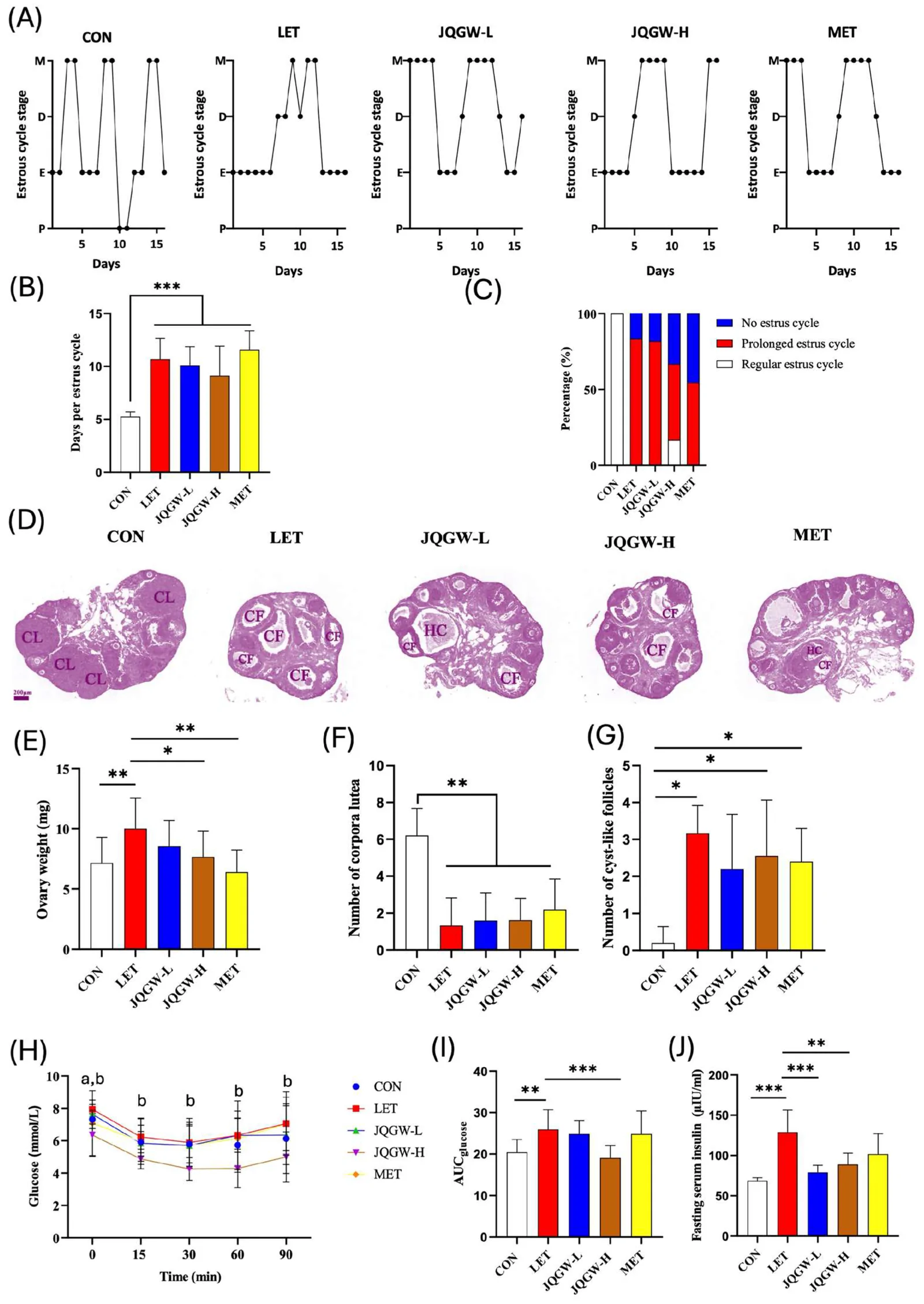
Establishment of the LET-induced PCOS-like mouse model. (**A**) Representative estrous cycles in each group of mice. M, metestrus; E, estrus; D, diestrus; P, proestrus. (**B**) Mean number of days for the estrous cycle in each group. (**C**) Percentage of a regular cycle, a long irregular cycle, and acyclicity in each group. (**D**) H&E staining in the ovary. CL indicates corpora lutea, CF indicates cystic follicles, and HC indicates hemorrhagic cysts. (**E**) Ovarian weight. (**F**) Average number of corpora lutea per ovary. (**G**) Average number of atretic cyst-like follicles per ovary. (**H**) Insulin tolerance test results in each group. The “a” label denotes that the CON group and the LET group are significantly different. The “b” label denotes that the LET group and the JQGW-H group are significantly different. (**I**) The AUC_glucose_ in each group. (**J**) The serum FINS levels in each group. The results are presented as the mean ± SEM (*n* = 6). * *p* < 0.05, ** *p* < 0.01, *** *p* < 0.001.

Control mice experienced various stages of the estrous cycle (estrus, metestrus, proestrus, or diestrus) over the course of 4–6 days, and female mice in the LET group needed a longer time to complete an estrous cycle (Figure 1B). When comparing the low-dose JQGW (JQGW-L), high-dose JQGW (JQGW-H), and metformin (MET) groups to the letrozole (LET) group, there were no appreciable differences in the number of days of the estrous cycles or in the number of completed estrous cycles in 16 days.

An increase in ovary weight has been observed among women with PCOS [21] and, in this study, LET treatment induced a marked increase in ovary weight compared to the control (CON) group. Conversely, the application of a high dose of JQGW along with MET protected against the increase in ovarian weight (Figure 1E). Meanwhile, oligo/anovulation is a crucial feature of PCOS relating to reproduction. According to histological analysis, the number of corpora lutea was significantly lower in the LET group, indicating the presence of oligo/anovulation (Figure 1D). No atretic cyst-like follicles were observed in the ovaries of the CON group. In contrast, the incidence of cystic follicles was markedly increased in all mice in the LET group compared to the CON group (Figure 1G). Both the JQGW-L and JQGW-H treatment groups exhibited a greater number of corpora lutea relative to the LET group. Cystic follicle numbers in the JQGW-L group were not significantly different from those in the MET group.

As shown in Figure 1H–J, the mice in the LET group had significantly reduced insulin tolerance, as indicated by significant increases in the glucose area under the curve (AUC_glucose_) and in fasting insulin (FINS). JQGW administration significantly alleviated insulin resistance in PCOS-like mice, and mice in the JQGW-L group had lower FINS levels than those in the LET group. In the JQGW-H group, both FINS and AUC_glucose_ were significantly decreased. No substantial improvement in insulin sensitivity was observed in either the MET or LET groups. In addition, LET-treated mice exhibited significantly increased body weight compared to CON mice (Supplementary Figure S4), indicating an obesity-prone sub-phenotype. This obesity-prone sub-phenotype was reversed by JQGW-H treatment.

### 2.2. JQGW Restored Endometrium Morphology and Function in LET-Induced PCOS-like Mice

Uterine factors are responsible for poor reproductive results in PCOS patients, and they have an elevated incidence of implantation failure even in those who experience successful ovulation [7]. To explore this further, we examined the uterine morphology in each female mouse. Uterine weight was significantly reduced in the LET group compared to the CON group. However, treatment with either JQGW or MET did not result in a notable restoration of uterine weight.

**Figure 2:**
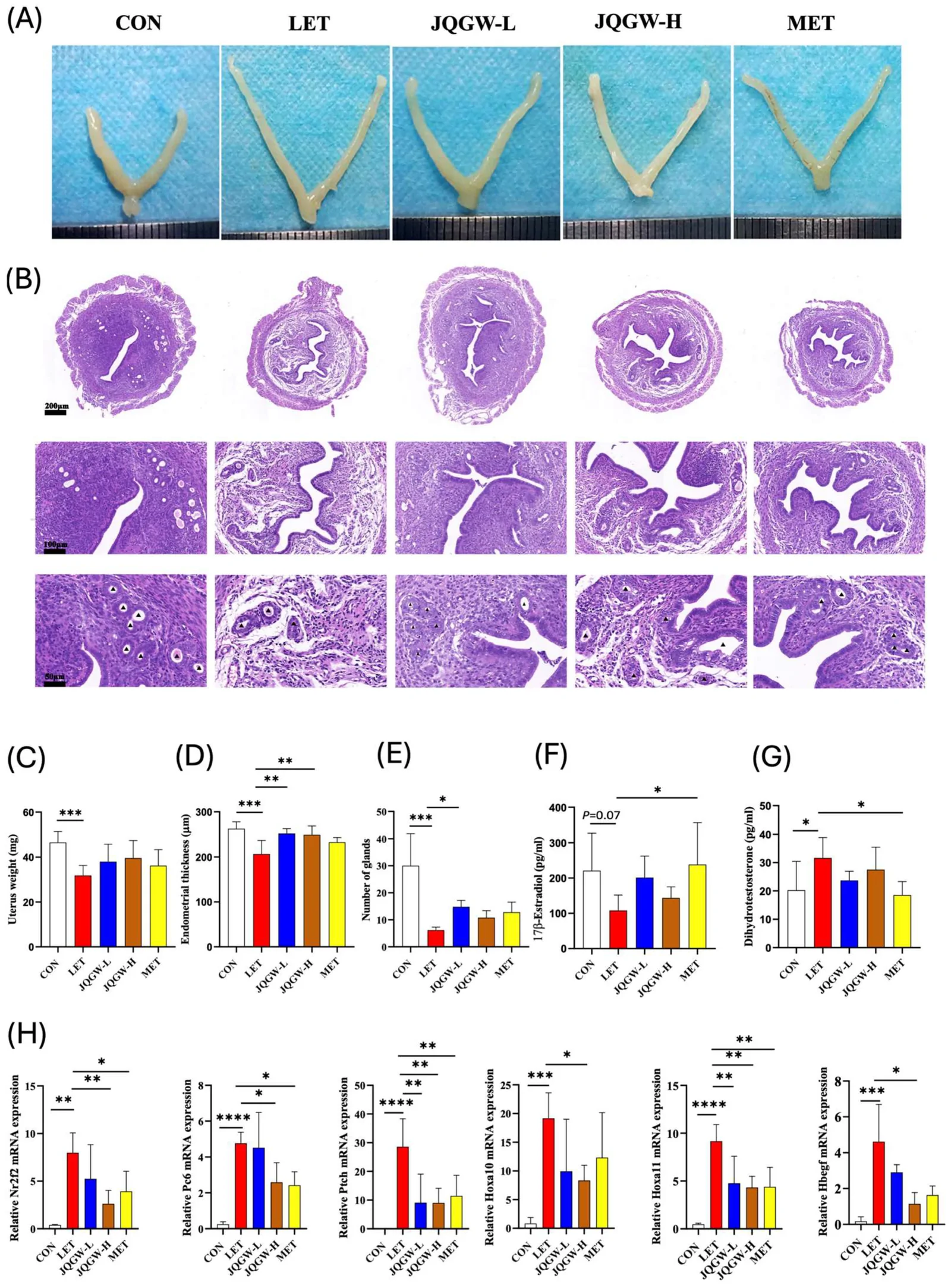
JQGW restored uterine morphology and functions in LET-induced PCOS-like mice. (**A**) Representative images of the uterus in each group. (**B**) H&E staining of the uterus. (**C**) Uterus weight in each group. (**D**) Average endometrial thickness. (**E**) Number of glands in the uterus. (**F**) Serum E2 concentrations in each group. (**G**) Serum DHT concentrations in each group. (**H**) Endometrial receptivity and decidualization-related genes, including *Nr2f2*, *Pc6*, *Ptch*, *Hoxa10*, *Hoxa11*, and *Hbegf*. The results are presented as the mean ± SEM (*n* = 5). * *p * <  0.05, ** *p*  <  0.01, *** *p*  <  0.001, **** *p* < 0.0001. Black triangles indicate the endometrial glands in the H&E-stained sections.

A prerequisite for endometrial receptivity is adequate endometrial thickness [22], and endometrial glands play a vital role in maintaining the implantation window, thus providing conditions for effective decidualization, uterine receptivity, and successful implantation [23]. To evaluate endometrial morphology, both the endometrial thickness and glandular density were measured in each female mouse. The LET group showed a significant decrease in endometrial thickness and a reduced number of epithelial and secretory glands compared to the CON group (Figure 2B). In the JQGW-L group, histological examination revealed a thicker endometrial lining and a significantly increased number of uterine glands compared to the LET group. No significant differences in endometrial thickness or gland count were detected between the MET and LET groups.

Endometrial receptivity and decidualization gene expression were further investigated (Figure 2H). The qPCR results showed increased expression of *Nr2f2*, *Pc6*, *Ptch*, *Hoxa10*, *Hoxa11*, and *Hbegf* in the LET group compared to the CON group, and this was reversed with JQGW-H treatment. In the JQGW-L group, only *Ptch* and *Hoxa11* showed significant reductions. Similarly, the MET group exhibited significant downregulation of *Nr2f2*, *Pc6*, *Ptch*, and *Hoxa11* when compared to the LET group.

The experimental groups’ serum testosterone levels were not significantly different (*p* > 0.05) (Supplementary Figure S2). However, there was a near-significant trend (*p* = 0.07; Figure 2F) showing that the LET group’s 17β-estradiol (E2) concentrations were generally lower than those of the CON group. Considering this, we looked at serum dihydrotestosterone (DHT) levels, another androgen subtype, and found that LET treatment significantly increased blood DHT levels (Figure 2G). Although E2 levels rose and DHT levels declined in both the JQGW-L and JQGW-H groups compared to the LET group, the differences were not statistically significant. In contrast, the MET group had significantly increased E2 levels and reduced DHT levels.

### 2.3. JQGW Ameliorated Uterine Inflammation in the PCOS-like Mice Through the Akt2/NF-κB p65 Pathway

No significant differences were observed in total Akt2 or NF-κB p65 protein levels among the experimental groups. However, relative to the CON group, the LET group showed marked elevations in p-Akt2 and p-NF-κB p65, as well as in their phosphorylation ratios (p-Akt2/Akt2 and p-NF-κB p65/NF-κB p65). p-Akt2 and the p-NF-κB p65/NF-κB p65 ratio were notably lower in the JQGW-L group compared to the LET group. The JQGW-H group showed a significant reduction in both the p-Akt2 level and the p-Akt2/Akt2 ratio. Compared to the LET group, the MET group had significantly decreased p-NF-κB p65 expression.

**Figure 3:**
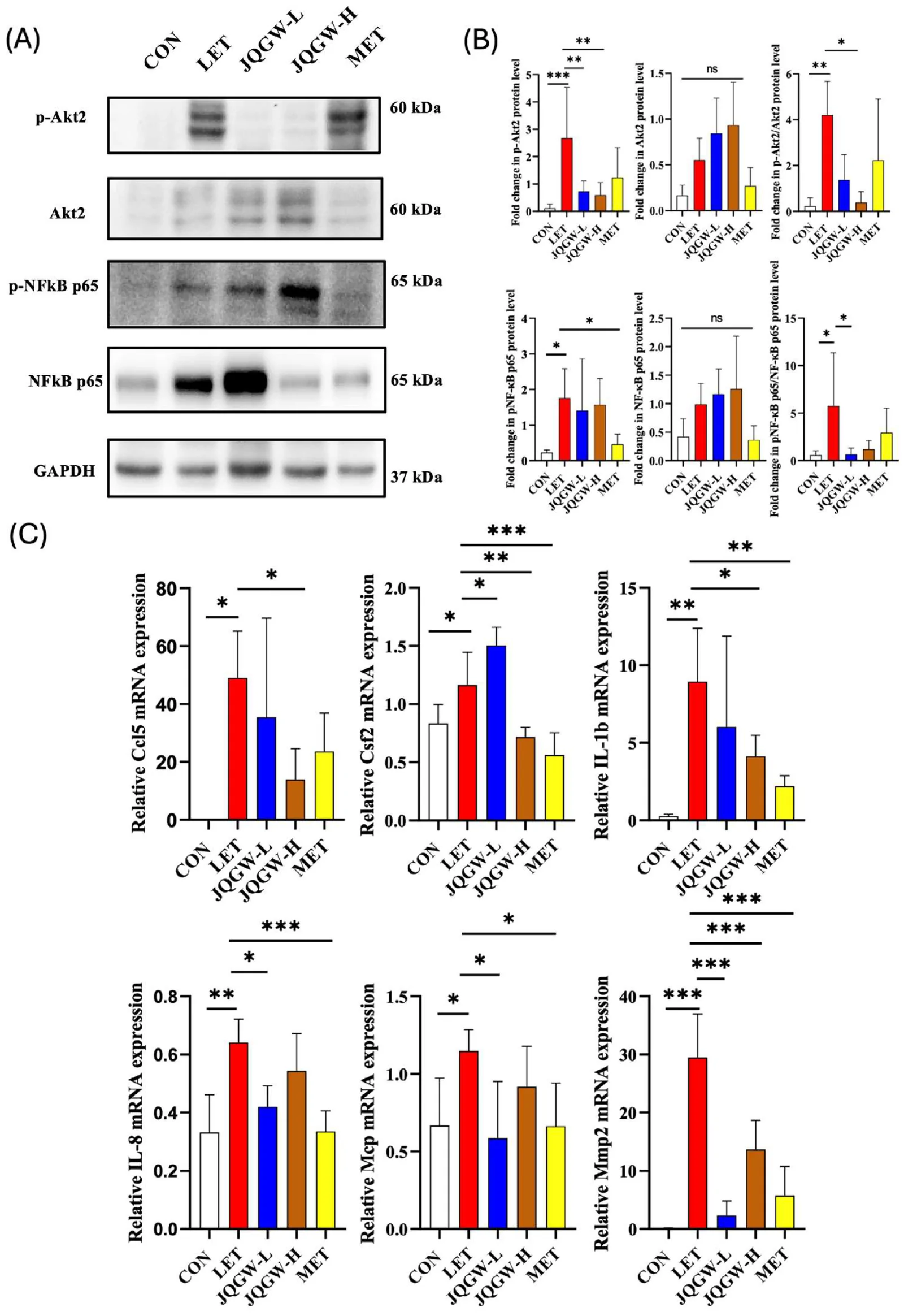
The effects of JQGW on the Akt2/NF-κB p65 signaling pathway. (**A**,**B**) Representative images and quantification of the densitometric data for p-Akt2, Akt2,p-NF-κB p65, and NF-κB p65. The relative protein levels of P-Akt2, Akt2, p-NF-κB p65, and NF-κB p65 and the p-Akt2/Akt2 and p-NF-κB p65/NF-κB p65 ratios. (*n* = 6/group) (**C**) The mRNA level of each gene is shown relative to the *Gapdh* mRNA level in the same sample. (*n* = 5/group). Values are expressed as the mean ± SEM. ns, not significant,* *p* < 0.05; ** *p* < 0.01; *** *p * <  0.001.

As Figure 3 illustrates, the mRNA levels of *Ccl5*, *Csf2*, *Il8*, *Il1b*, *Mcp-1*, and *Mmp2* were significantly increased in the LET group compared to the CON group. Treatment with JQGW-L considerably downregulated *Csf2*, *Il8*, *Il1b*, *Mcp-1*, and *Mmp2* mRNA levels compared to the LET group, and in the JQGW-H group, *Ccl5*, *Csf2*, *Il1b*, and *Mmp2* were significantly downregulated. The MET group also had a significant decrease in *IL-8*, *IL-1β*, *MCP-1*, and *Mmp2* mRNA levels compared to the LET group.

### 2.4. JQGW Partially Restored the Diversity of the Gut Microbiota in PCOS-like Mice

**Figure 4:**
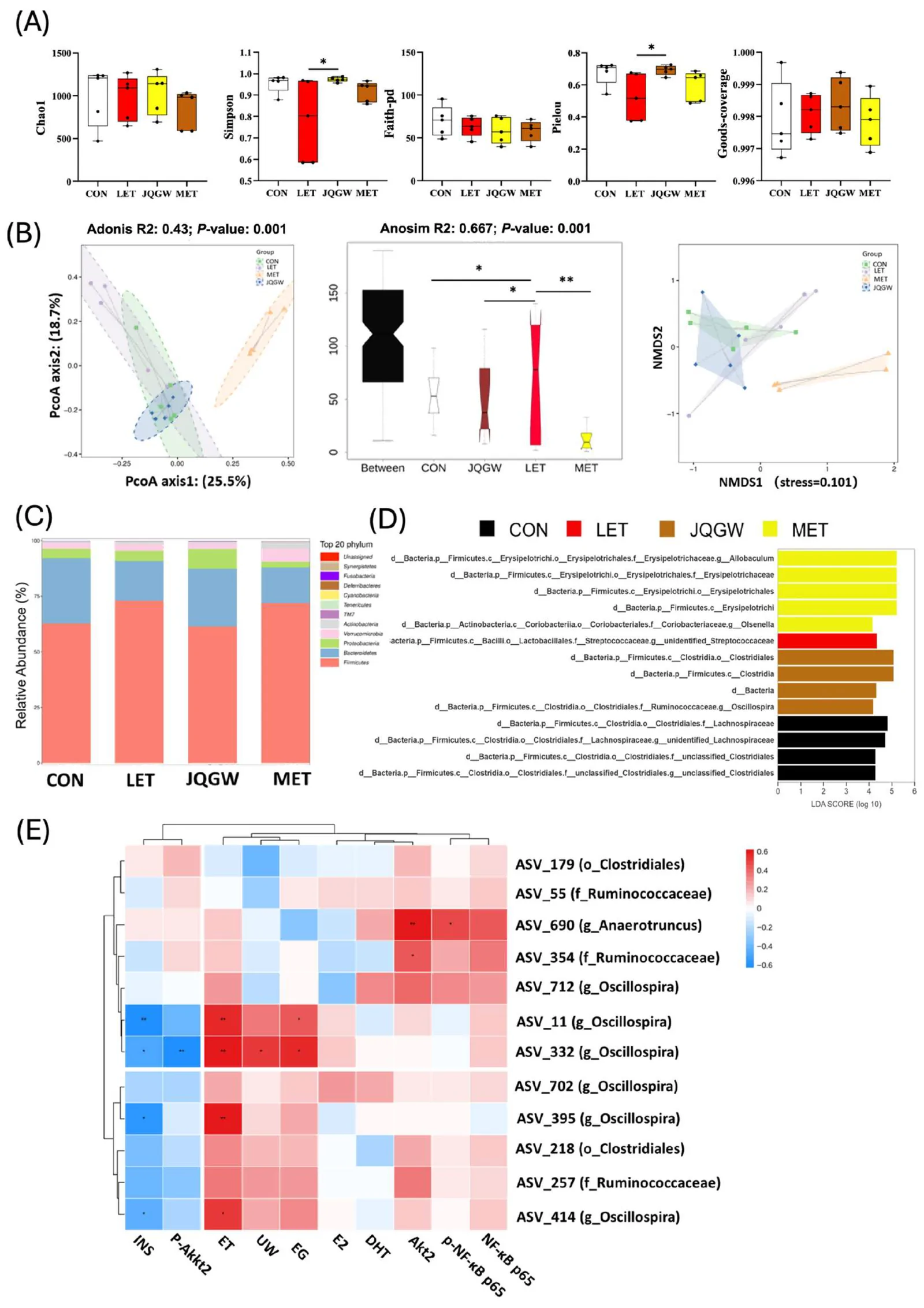
Effects of JQGW on the diversity of the gut microbiota in PCOS-like mice. (**A**) The α-diversity of gut bacterial assemblages: Chao1 index, Simpson index, Faith-pd index, Pielou index, and Goods-coverage index. (**B**) β-diversity analysis of the intestinal microbiota in different groups, including Bray–Curtis-based principal coordinates analysis (PCoA), pairwise ANOSIM results, and weighted UniFrac-based non-metric multidimensional scaling (NMDS). (**C**) The relative abundance of bacterial communities at the total phylum level. The stacked bars include all identified phyla; extremely rare taxa (relative abundance < 1%) are also presented but may appear as thin bands. (**D**) Linear discriminant analysis effect size analysis of different groups with a linear discriminant analysis (LDA) score > 4. (**E**) Heatmap of the Spearman correlation analysis between intestinal flora and uterine traits. Values are expressed as the mean ± SEM. * *p* < 0.05; ** *p* < 0.01.

The α-diversity indices in the LET group showed no significant differences compared to the CON and MET groups (Figure 4A). Remarkably, JQGW treatment significantly enhanced gut microbial diversity and uniformity, with elevated Simpson and Pielou indices relative to the LET group.

As shown in Figure 4B, Bray–Curtis-based principal coordinates analysis and weighted UniFrac-based non-metric multidimensional scaling (stress = 0.101) showed distinct clustering between the LET and CON groups, indicating substantial compositional divergence. With JQGW treatment, the gut microbial community structure shifted toward that of the CON group. Analysis of similarities (ANOSIM) confirmed that intergroup differences were statistically significant.

A total of 6994 amplicon sequence variations (ASVs) were identified across all samples (Supplementary Figure S3). The overlap between the CON and LET groups accounted for only 13.07% of all species, and the overlap between the CON and JQGW groups was relatively higher at 13.41%, indicating that the JQGW group’s microbiological profile was more like that of the CON group. The phylum-level gut microbial makeup is shown in Figure 4C, and Firmicutes, Bacteroidetes, Proteobacteria, Verrucomicrobia, and Actinobacteria were the most common phyla in all groups. Verrucomicrobia showed a trend of enrichment in the LET group relative to the CON group (*p* > 0.05), and the F/B ratio was elevated. JQGW treatment led to a lower Firmicutes/Bacteroidetes (F/B) ratio, while concurrently increasing Proteobacteria levels. At the family level, Lachnospiraceae was more prevalent in the CON group, whereas Streptococcaceae was enriched in LET-treated mice. Notably, *Oscillospira* abundance increased following JQGW administration, and Allobaculum levels were elevated in the MET group.

The analysis revealed significant correlations between specific microbial populations and uterine traits such as uterine weight, endometrial thickness, and the number of endometrial glands (Figure 4E). In addition, serum hormone levels (insulin, E2, and DHT) were also found to be closely linked to the gut microbiota composition. Among the identified taxa, *g_Oscillospira* (ASV_332) exhibited a positive correlation with all three uterine parameters while showing a negative correlation with serum insulin levels and uterine p-Akt2 protein expression. Similarly, *g_Oscillospira* (ASV_11) was positively linked with endometrial thickness and gland counts, but negatively linked with serum insulin levels. *g_Oscillospira* (ASV_395) and *g_Oscillospira* (ASV_414) were positively correlated with endometrial thickness and negatively correlated with serum insulin levels. Furthermore, *g_Anaerotruncus* (ASV_690) and *f_Ruminococcaceae* (ASV_354) had a favorable connection with uterine Akt2 protein levels.

### 2.5. JQGW Alleviated Serum Metabolomic Profile Changes in PCOS-like Mice

**Figure 5:**
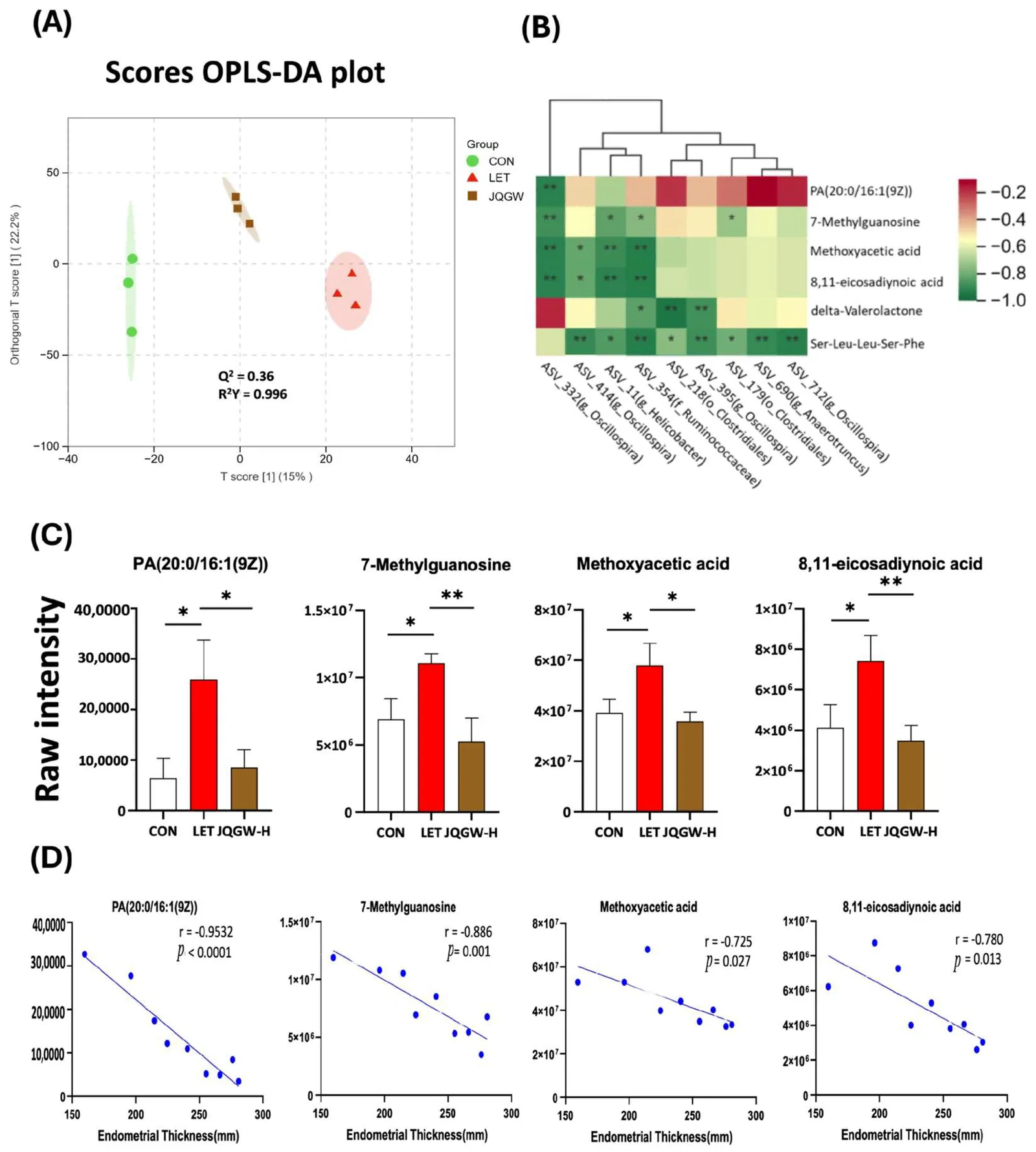
The effects of JQGW on serum metabolomic profiles in PCOS-like mice. (**A**) OPLS-DA results. (**B**) Spearman’s rank correlation heatmaps of the gut microbiota and differential serum metabolites. (**C**) The relative abundance of four metabolites among the three groups. (**D**) Pearson’s correlation results between the endometrial thickness and the four metabolites. Each dot represents one subject; the blue line indicates the linear regression fit; r and *p* values are from Pearson correlation analysis. Values are expressed as the mean ± SEM. * *p* < 0.05; ** *p* < 0.01.

A non-targeted metabolomic analysis was conducted to quantify serum metabolites in the CON, LET, and JQGW-H groups, aiming to investigate potential links between gut microbial composition and host metabolic profiles. For the OPLS-DA model, Q^2^ = 0.36, R^2^X = 0.376, and R^2^Y = 0.996 (Figure 5A). As a general rule, a model is considered credible if 0.3 < Q^2^ ≤ 0.5, and thus, the Q^2^ values of our samples indicated that the OPLS-DA model was credible [24]. Although a Q^2^ value of 0.36 is within an acceptable exploratory range reported in some biological metabolomics studies, this threshold is admittedly less widely accepted than the standard Q^2^ > 0.5. Furthermore, due to the limited sample size (*n* = 3 per group), independent validation via permutation testing was not performed. These factors represent a limitation in the validation and predictive power of the current OPLS-DA model, though the data still provide valuable exploratory insights.

As shown in Table 1, the relative abundances of 11 serum metabolites were compared across the three groups. The LET group exhibited markedly altered metabolite profiles compared to the CON group. Notably, administration of JQGW in PCOS-like mice resulted in observable shifts in the abundance of these metabolites (*p* < 0.05; q > 0.05, see Table 1). Given the exploratory nature of this discovery-based metabolomics analysis and the conservative FDR correction, these findings should be considered hypothesis-generating and require targeted validation with larger sample sizes and authentic standards.

Nine bacterial species and six typical metabolites were used to further investigate the gut–metabolite axis (Figure 5B). Notably, *g_Oscillospira* (ASV_332) was negatively correlated with PA (20:0/16:1(9Z)), 7-Methylguanosine, Methoxyacetic acid, and 8,11-Eicosadiynoic acid. Similar negative associations were observed for *g_Oscillospira* (ASV_414 and ASV_11) with additional metabolites, including Ser-Leu-Leu-Ser-Phe, and Glycerophospho-N-Palmitoyl Ethanolamine. *f_Ruminococcaceae* (ASV_354) showed negative correlations with eight metabolites, while *o_Clostridiales*, *g_Anaerotruncus* and other *Oscillospira* variants also exhibited consistent inverse relationships with key metabolic markers. Levels of PA (20:0/16:1(9Z)), 7-Methylguanosine, Methoxyacetic acid, and 8,11-Eicosadiynoic acid were higher in the LET group compared to the CON group (*p* < 0.05 or *p* < 0.01; Figure 5C). Administration of JQGW-H effectively lowered the concentrations of these metabolites, restoring them to baseline levels.

To assess the relevance of these metabolites to uterine function, Pearson’s correlation analysis was performed. All four metabolites demonstrated strong negative correlations with endometrial thickness (Figure 5D). However, given that none of these metabolites passed FDR correction (q > 0.05, Table 1), these correlations should be interpreted cautiously and considered exploratory. Among the identified metabolites, PA (20:0/16:1(9Z)) showed the highest negative connection with uterine parameters (r = −0.9352, *p* < 0.001), followed by 7-Methylguanosine (r = −0.886, *p* < 0.001), 8,11-Eicosadiynoic acid (r = −0.780, *p* < 0.01), and Methoxyacetic acid (r = −0.725, *p* < 0.01) (Figure 5D).

## 3. Discussion

### 3.1. JQGW Improves Metabolic and Uterine Features in PCOS-like Mice

This research demonstrated the efficacy of JQGW in the PCOS mouse model. JQGW treatment significantly reduced ovarian weight, hyperglycemia, and hyperinsulinemia, but not the number of corpora lutea or cystic follicles in PCOS-like mice. JQGW increased endometrial thickness and gland numbers and restored these receptivity markers toward their normal levels. Additionally, JQGW reduced inflammation in the endometrium by blocking the Akt2/NF-κB p65 signaling pathway. In addition, JQGW restored the gut microbiota’s β-diversity, with significant enrichment of Oscillospira. Notably, the abundance of Oscillospira (ASV_332) was positively associated with uterine weight, endometrial thickness, and gland number. In addition, metabolites like PA (20:0/16:1(9Z)), 7-Methylguanosine, Methoxyacetic acid, and 8,11-Eicosadiynoic acid were significantly elevated in PCOS-like mice and showed negative correlations with both Oscillospira (ASV_332) abundance and endometrial thickness.

### 3.2. JQGW Restores Endometrial Morphology and Receptivity

Endometrial impairment is a hallmark of PCOS-related infertility, manifesting as reduced endometrial thickness and reduced glandular development. In our study, significant reductions in both parameters were seen in the PCOS-like mice, and this was consistent with impaired implantation capacity [25,26]. JQGW administration restored the endometrial architecture, suggesting enhanced receptivity. Molecular analysis revealed the upregulation of the receptivity-related genes *Nr2f2*, *Pc6*, *Ptch*, and *Hbegf* in the LET group, which was reversed by JQGW. In a previous study, *Nr2f2*, *Pc6*, *Ptch*, and *Hbegf* gene expression was found to be increased in the uterus of pregnant PCOS-like rats [27]. Although HOX gene expression is typically downregulated in PCOS [28,29], our findings align with reports linking elevated HOXA10 and IL1β expression to obesity-related PCOS phenotypes [30], indicating that body weight might influence gene regulation in this model (Supplementary Figure S4). The paradoxical upregulation of *Hoxa*10 and *Hoxa*11 in LET-treated mice, contrary to most PCOS literature, warrants careful interpretation. The increased body weight observed in LET mice (Supplementary Figure S4) suggests an obesity-prone sub-phenotype. Consistent with a previous report linking elevated HOXA10 and IL1B to overweight PCOS patients [30], the upregulation in our LET mice may reflect obesity-related inflammation rather than a true functional receptivity program. It should be noted that metformin, the first-line pharmacological treatment for PCOS, did not significantly improve insulin sensitivity or endometrial thickness in this study. While unexpected, this finding does not invalidate the model. Metformin’s primary effects are on metabolic parameters, and its direct impact on endometrial receptivity is less consistently reported. The dose (750 mg/kg/day) or treatment duration (35 days) may have been suboptimal for uterine-specific outcomes in our mouse model. Importantly, we do not conclude that JQGW is superior to metformin; rather, our results suggest that JQGW exerts beneficial effects on uterine endpoints under conditions where metformin did not, which may reflect differences in their mechanisms of action (e.g., gut microbiota modulation).

### 3.3. JQGW Suppresses Endometrial Inflammation via the Akt2/NF-κB Pathway

In PCOS, subclinical endometrial inflammation triggers the NF-κB signaling pathway [31]. In addition, members of the PI3K/Akt pathway, particularly Akt2, play a central role in modulating NF-κB activity and balancing apoptosis and proliferation during endometrial preparation [32,33]. Akt2, a mitochondrial-localized isoform of Akt, is implicated in glucose metabolism and cell survival [34], and its genetic variants have been linked to increased susceptibility to PCOS [35]. PCOS patients’ endometrium has been shown to have an overactive PI3K/Akt pathway [36], but the specific expression patterns of Akt2 in uterine tissue remain unexplored. In this study, the activation of inflammatory signaling via the Akt2/NF-κB pathway was predominantly observed in the uterine tissue of LET-treated mice. These changes are consistent with previous reports of heightened NF-κB activity in PCOS patients’ endometrium [37,38]. JQGW treatment significantly suppressed Akt2/NF-κB activation and reduced inflammatory gene expression, indicating that its efficacy might be due to the regulation of this pathway.

### 3.4. JQGW Modulates Gut Microbiota Composition

The gut microbiota maintains the intestinal barrier’s integrity and controls physiological processes, including metabolism [39]. Dysbiosis disrupts this balance and increases proinflammatory factors like lipopolysaccharide, thus contributing to intestinal damage and systemic inflammation [40]. Gut dysbiosis has been linked to various reproductive disorders, including PCOS [41], and in PCOS, dysbiosis is reflected by altered α and β-diversity, an imbalance in the F/B ratio, and changes in bacterial taxa abundance [42], and this suggests a role for IL-22 and bile [43]. Distinct β-diversity shifts were found between the CON and LET groups, and these changes were consistent with previous findings in PCOS models [44]. Streptococcaceae was found to be a potential biomarker for PCOS in mice in this study, consistent with its elevated abundance in PCOS patients [45,46].

TCM herbs interact with the gut microbiota, thus altering microbial composition and producing bioactive or toxic metabolites through microbial fermentation [47]. JQGW restored the microbial composition to that of the CON group, showing reduced F/B ratio and increased Proteobacteria abundance. Notably, *Oscillospira*, identified as a gut microbial biomarker in the JQGW group, was negatively correlated with serum insulin levels, suggesting that JQGW may alleviate insulin resistance by increasing the abundance of *Oscillospira*. Despite the lack of pure culture isolation, *Oscillospira* has consistently emerged in high-throughput sequencing studies as a beneficial genus in metabolic and chronic inflammatory diseases [48,49]. Its ability to produce SCFAs and its association with gut homeostasis support its potential as a next-generation probiotic candidate [49].

*Oscillospira* was also positively correlated with uterine weight, endometrial thickness, and gland number. Infertile women have been found to exhibit lower relative abundances of vaginal *Oscillospira* compared to fertile women [50], but few studies have reported an association between *Oscillospira* and uterine function. Generally, gut microbiota may improve reproductive function through two primary mechanisms [51], namely by modulating host hormone levels, such as systemic estrogen via the estrobolome [52], and by producing microbial-derived metabolites, including SCFAs [53,54], which influence endometrial inflammation, cytokine expression, and tissue remodeling through interactions with host receptors and signaling pathways [55]. Here, we found that serum insulin levels but not E2 or DHT levels had a significant correlation with microbial biomarkers in the gut in the JQGW-H group. Excess insulin disrupted glucose transporter type 4 (GLUT4) in the endometrial stroma [56]. MET, which is commonly prescribed when lifestyle modifications are inadequate [57], alleviates insulin resistance by modulating multiple signaling pathways, including GLUT4, to inhibit gluconeogenesis, thereby contributing to improved endometrial health in women with PCOS [58]. Therefore, we suggest that *Oscillospira* enhances endometrial receptivity by reducing insulin resistance.

### 3.5. JQGW Alters Serum Metabolite Profiles Linked to Uterine Function

Beyond universal metabolites, specific metabolites originating from the gut microbiota also contribute to reproductive system disorders [51]. For instance, the microbial metabolite agmatine activates the farnesoid X receptor and thereby inhibits glucagon-like peptide-1 to promote PCOS in female mice [59]. In this study, four metabolites—including PA (20:0/16:1(9Z)), 7-Methylguanosine, Methoxyacetic acid, and 8,11-Eicosadiynoic acid—were elevated in LET-treated mice and were negatively correlated with *Oscillospira* (ASV_332) abundance and endometrial thickness. JQGW-H treatment significantly reduced their serum levels. PA (20:0/16:1(9Z)) belongs to the class of glycerophospholipids, and as the main lipid components in mammalian cell membranes, glycerophospholipids are vital for controlling transport, cell signaling, and protein activity [60]. Alterations in glycerophospholipid profiles have been reported in the follicular fluid of PCOS patients in contrast to healthy individuals [61,62]. 7-Methylguanosine (m7G) is a modified nucleoside found in tRNA and as the 5′ cap structure (m7GpppN) of eukaryotic mRNAs. However, the free 7-methylguanosine measured in serum is chemically distinct from the cap structure and is thought to derive primarily from RNA turnover, particularly tRNA degradation. Consistent with this, a recent study reported significantly increased free 7-methylguanosine levels in the peripheral blood of PCOS patients compared to healthy controls [63]. Methoxyacetic acid has reproductive and developmental toxicity [64], and it can directly cause pachytene spermatocyte toxicity [65]. In addition, 8,11-Eicosadiynoic acid, which belongs to the family of polyunsaturated fatty acids, has been shown to inhibit eicosanoid biosynthesis and cyclooxygenase activity [66,67]. These metabolites are associated with membrane signaling, RNA methylation, and reproductive toxicity. Their reduction following JQGW treatment suggests that JQGW may help alleviate PCOS-related uterine impairment by modulating the serum metabolite profiles linked to inflammation and cellular stress. Whether these metabolites can directly improve the endometrial environment in PCOS remains to be further investigated. It should be noted that none of these four metabolites passed FDR correction (q > 0.05, Table 1), which is likely attributable to limited statistical power. Therefore, these findings are exploratory and hypothesis-generating, and require validation in future studies with larger sample sizes.

### 3.6. Strengths and Limitations

This study has several strengths. It integrates endometrial functional assessments with 16S rRNA sequencing and untargeted metabolomics, providing a systems-level perspective on the gut–uterus axis. The use of a well-established letrozole-induced PCOS mouse model, multiple treatment doses, and a positive control (metformin) strengthens the validity of the findings. The identification of *Oscillospira* as a potential microbial biomarker associated with uterine recovery offers a novel target for future interventions.

Several limitations should be acknowledged. First, the absence of functional reproductive assessments, such as mating and pregnancy outcomes, limits the direct translation of endometrial improvements to increased fertility. Second, only correlations between gut microbiota, metabolites, and uterine parameters were observed, and causal relationships remain to be established. Third, from a methodological perspective, the ultrasonication-assisted dissolution used in this study may alter the chemical profile compared to the standard clinical preparation (hot water dissolution without ultrasonication), and compound identifications are tentative as no reference standards were used for confirmation. Fourth, the metabolomics findings did not withstand FDR correction. This is likely due to the modest sample size and biological variability inherent to the PCOS-like model. Therefore, these results should be interpreted as exploratory. Future studies with larger cohorts and targeted quantitative approaches (e.g., LC-MS/MS with authentic standards) are needed to validate the observed trends. Collectively, future studies should validate these findings using fertility-based outcomes and establish causal links through microbiota transplantation or metabolite supplementation experiments. For metabolomics analysis, only 3 mice per group were analyzed due to insufficient residual serum volume after other measurements. This sample size is below the recommended minimum for untargeted metabolomics studies, which typically require at least 6 animals per group to ensure adequate statistical power.

## 4. Materials and Methods

### 4.1. Quality Control of JQGW and the Preparation of Reagents

Granules of the TCM formula investigated in this study were purchased from Yata Pharmaceutical Co., Ltd, Shaoxing, Zhejiang, China. Batch-specific information is maintained by the manufacturer and can be made available upon request. The crude drugs were authenticated by hospital pharmacists in accordance with the Chinese Pharmacopoeia (2020 edition). Briefly, the starting material was clinically used formula granules of Banxia (*Pinellia peltata* C.Pei, 1.5 g), Chenpi (*Citrus reticulata* Blanco, 2 g), Fuling (*Wolfiporia extensa* (Peck) Ginns, 1 g), Chuanxiong (*Ligusticum chuanxiong* S.H.Qiu, Y.Q.Zeng, K.Y.Pan, Y.C.Tang & J.M.Xu, 2.6 g), Xiangfu (*Cyperus rotundus* L., 1 g), Baizhu (*Atractylodes carlinoides* (Hand.-Mazz.) Kitam., 6 g), Cangzhu (*Atractylodes lancea* (Thunb.) DC., 2.2 g), Shenqu (Massa Medicata Fermentata, 0.6 g), and Gancao (*Glycyrrhiza uralensis* Fisch. ex DC., 1 g). The granules were immersed in hot water (heated to 100 °C) and subjected to ultrasonication at 95 °C for 90 min [20]. It should be noted that ultrasonication was applied solely to aid dissolution and to ensure the complete release of chemical components for analytical purposes and does not represent a standard “extraction” procedure as used for raw herbs. This preparation method differs from the clinical administration method (simple dissolution in hot water without ultrasonication) and may therefore alter the chemical profile compared to the actual clinical preparation. Compound identification was performed by matching the acquired MS/MS spectra against databases (e.g., TCMSP, PubChem & ChemSpider). All identifications are tentative, based on retention time, MS fragmentation patterns, and comparison with database. No reference standards were used for confirmation. Representative chromatograms and the tentatively identified compounds are presented in Supplementary Table S1 and Supplementary Figure S1.

### 4.2. Animals and Treatment Groups

**Figure 6:**
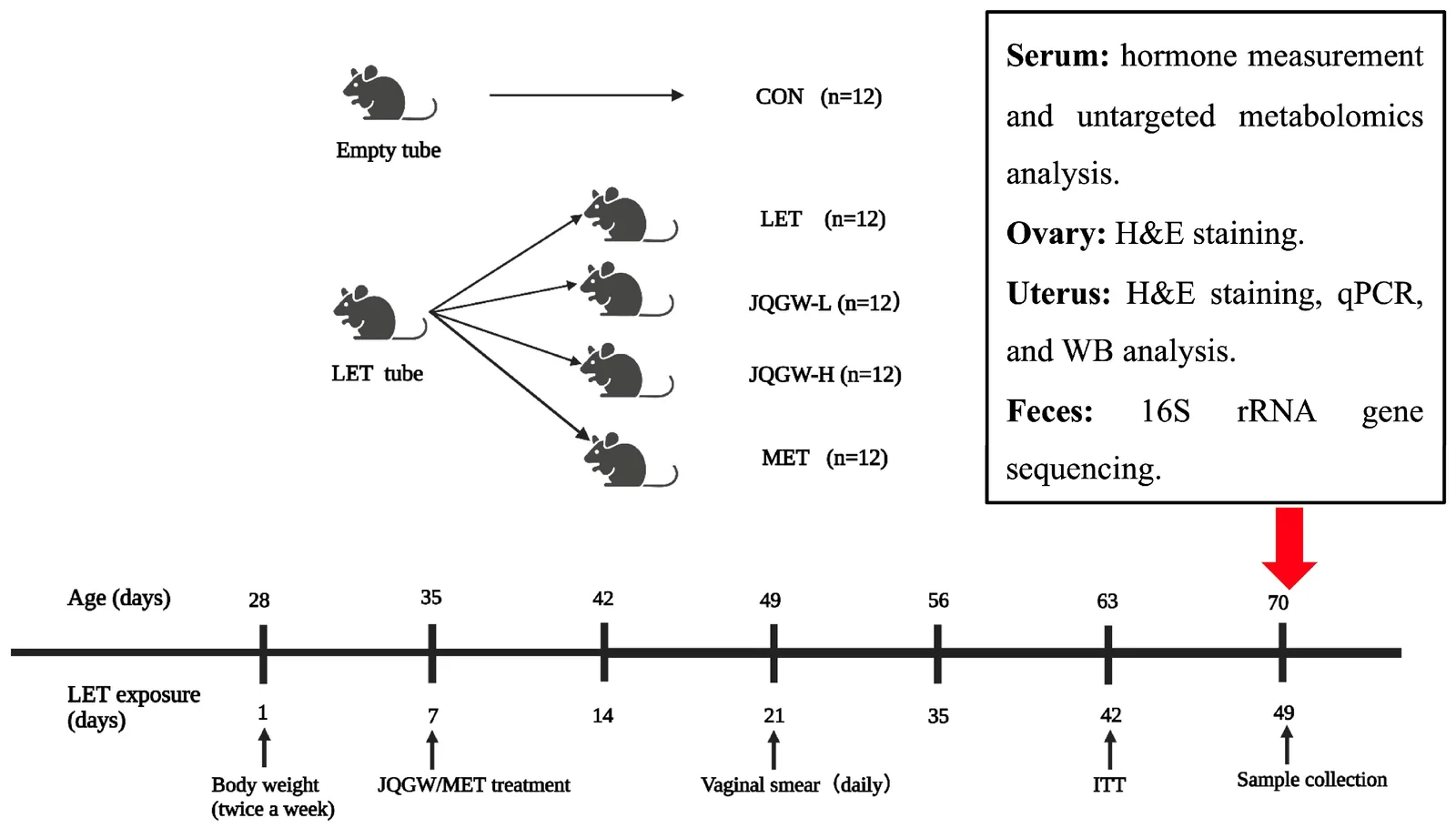
Experimental procedures.

All animal experiments were approved by the Institutional Animal Care Board (Approval No. 2021304) on 27 July 2021. A total of 60 female C57BL/6J mice, aged 21 days (11–13 g), were randomly assigned into five experimental groups (*n* = 12 per group): control (CON), letrozole-induced model (LET), low-dose JQGW treatment (JQGW-L), high-dose JQGW treatment (JQGW-H), and MET-treated (MET). PCOS was induced by subcutaneous implantation of silastic tubes (8 mm; ID 0.04 mm, OD 0.085 mm; Dow Corning, Midland, MI, USA) containing 4.5 mg LET (Shanghai Macklin Biochemical Technology Co., Shanghai, China) following established protocols (Xue et al., 2018) [67]. Mice in the CON group received blank implants. Beginning on day 35 post-implantation, mice received daily oral administration for a duration of 35 days. Treatment regimens included low-dose JQGW (23.8 g/kg/day), high-dose JQGW (47.6 g/kg/day), and MET (750 mg/kg/day; Sigma-Aldrich, St. Louis, MO, USA), each formulated in 0.2 mL of distilled water prior to gavage. Body weight was monitored twice weekly, and vaginal smears were collected daily from days 49 to 64 to assess estrous cycles. After 35 days of treatment, mice were fasted for 6 h and subsequently anesthetized with isoflurane (RWD Life Science, Shenzhen, China). Serum samples, ovaries, uteruses, and the ileocecal contents were collected. All mice were sacrificed during the diestrus stage of the estrous cycle, as determined by daily vaginal smears. Serum samples were preserved at −80 °C, while tissue specimens were processed either by fixation in 4% paraformaldehyde (Biosharp, Hefei, China) for histopathological examination or immediately snap-frozen for subsequent microbiome and molecular analyses. Sample sizes for each analysis: All 12 mice per group were used for non-terminal measurements (body weight, estrous cycle, insulin tolerance test). 6 mice per group were randomly selected for histology and Western blot. For qRT-PCR and 16S rRNA sequencing, one sample was excluded due to poor RNA integrity, leaving 5 mice per group. For serum metabolomics, only 3 mice per group had sufficient residual serum volume after hormone assays (≥100 µL) and were included. The experimental design is illustrated in Figure 6.

Sixty female C57BL/6J mice (21 days old) were equally and randomly divided into five experimental groups (*n* = 12/group): control (CON), letrozole-induced model (LET), low-dose JQGW treatment (JQGW-L), high-dose JQGW treatment (JQGW-H), and MET-treated (MET). PCOS was induced by subcutaneous implantation of tubes containing 4.5 mg LET. Mice in the CON group received blank implants. Beginning on day 35 post-implantation, mice received daily oral administration for a duration of 35 days. Treatment regimens included low-dose JQGW (23.8 g/kg/day), high-dose JQGW (47.6 g/kg/day), and MET (750 mg/kg/day). Body weight was monitored twice weekly, and vaginal smears were collected daily from days 49 to 64 to assess estrous cycles. After 35 days of treatment, the mice were sacrificed, and tissues were collected.

### 4.3. Insulin Tolerance Tests (ITTs)

After a 4 h fast, intraperitoneal injections of 0.5 U/kg of insulin were used to perform the ITTs. An Accu-Chek glucometer (Roche, Indianapolis, IN, USA) was used to measure blood glucose levels at predetermined intervals (0, 30, 60, and 90 min). Blood was taken from the tip of the tail.

### 4.4. Hematoxylin and Eosin (H&E) Staining

Ovarian and uterine samples were immersed in 4% paraformaldehyde for 48 h, followed by standard paraffin embedding procedures for subsequent histological preparation. Maximum longitudinal sections of the ovary and transverse sections of the uterus were prepared at a thickness of 4 μm and subjected to H&E staining for histological evaluation. Morphological analysis included quantification of cystic follicles and corpora lutea in the ovaries, as well as measurement of endometrial thickness and quantification of endometrial glands. Six mice per group were used for histological assessments.

### 4.5. Measurement of Serum Biochemical Markers

Serum testosterone (Cloud-Clone, Corp., Katy, TX, USA), DHT (R&D Systems, Inc., Minneapolis, MN, USA), E2 (Cayman Chemical Co., Ann Arbor, MI, USA), and insulin (RayBiotech, Inc., Peachtree Corners, GA, USA) were quantified. All assays were performed in strict accordance with the manufacturers’ instructions. Six mice per group were employed for biochemical analysis.

### 4.6. Quantitative Real-Time PCR (qRT-PCR)

Uterine tissues from mice were processed for total RNA isolation with a commercially available kit. cDNA was generated through reverse transcription, followed by qRT-PCR using SYBR Green dye in accordance with the supplier’s protocol. Expression data were normalized against *Gapdh* and analyzed using the 2^−ΔΔCt^ approach. Primer details are provided in Table 2.

### 4.7. Western Blot Analysis

Uterine proteins were extracted using RIPA buffer supplemented with protease and phosphatase inhibitors following tissue homogenization. All procedures were conducted on ice to maintain protein stability. Each sample containing 20 μg of protein was resolved by SDS-PAGE and subsequently transferred to PVDF membranes. The membranes were then incubated in 5% nonfat milk for 2 h at ambient temperature to block nonspecific binding and then incubated overnight at 4 °C with primary antibodies targeting phospho-Akt2 (Ser474) (#8599, CST), Akt2 (#2964, CST), phospho-NF-κB p65 (#3033, CST), and total NF-κB p65 (#2118, CST). These primary antibodies were all rabbit-derived and diluted 1:1000. Membranes were incubated with secondary antibodies (goat anti-rabbit IgG or horse anti-mouse IgG, 1:2000 dilution) for 2 h at room temperature. Protein bands were visualized using chemiluminescent reagents and quantified relative to GAPDH (#5174, CST) expression.

### 4.8. Gut Microbiome Analysis

Fecal samples were collected aseptically and stored at −80 °C prior to DNA extraction. Genomic DNA quality was assessed by 1.2% agarose gel electrophoresis. The V3–V4 region of the bacterial 16S rRNA gene was amplified using primers 338F (5′-ACTCCTACGGGAGGCAGCA-3′) and 806R (5′-GGACTACHVGGGTWTCTAAT-3′). The resulting amplicons were purified, quantified, and sequenced on the Illumina MiSeq platform (2 × 300 bp paired-end reads). Quality-checked reads were processed using the DADA2 pipeline in QIIME2(version 2024.5), generating amplicon sequence variants (ASVs). Taxonomy was assigned using the Greengenes database (Release 13.8). LEfSe analysis (LDA > 2.0, *p* < 0.05) was used to identify differentially abundant taxa.

### 4.9. Untargeted Metabolomics

Serum specimens were defrosted on ice and processed with an acetonitrile–methanol mixture (1:4, *v*/*v*) supplemented with internal standards. After centrifugation, the supernatants were analyzed by liquid chromatography–mass spectrometry (LC-MS) under both positive and negative ionization modes. Chromatographic separation was performed on a Waters ACQUITY Premier HSS T3 column (1.8 μm, 2.1 mm × 100 mm, Waters Corporation, Milford, MA, USA) at 40 °C. The mobile phase consisted of (A) 0.1% formic acid in water and (B) 0.1% formic acid in acetonitrile. The gradient program was as follows: 0–2 min, 5–20% B; 2–5 min, 20–60% B; 5–12 min, 60–95% B, followed by re-equilibration. The flow rate was 0.4 mL/min, and the injection volume was 4 μL. Mass spectrometry was performed on a Thermo Scientific Q Exactive HF-X Orbitrap mass spectrometer coupled to a Vanquish UHPLC system (both from Thermo Fisher Scientific, Waltham, MA, USA), operating in positive and negative electrospray ionization modes over a mass range of *m*/*z* 75–1000. Mass spectra data were acquired over an *m*/*z* range of 75–1000. Raw data were converted to mzXML format and processed using XCMS (version 3.18.0) in R (version 4.4.1). Metabolite identification was achieved by matching spectral features against publicly available databases.

### 4.10. Statistical Analysis

Statistical analyses were performed using GraphPad Prism (version 6) and SPSS (version 25). Given the small sample size (*n* = 5–6 per group), formal normality testing has limited power. Therefore, normality was assessed by visual inspection of Q-Q plots and histogram distributions. For data that appeared approximately normally distributed, one-way ANOVA with Tukey’s post hoc test was used. For non-normal or heteroscedastic data, the Kruskal–Wallis test with Dunn’s post hoc test was applied. For normally distributed data, parametric tests including the independent samples *t*-test and one-way ANOVA with Tukey’s post hoc were applied. Non-parametric alternatives, such as the Mann–Whitney U-test and Kruskal–Wallis test, were used for non-normal data. Repeated-measures ANOVA was employed to evaluate body weight and ITT results, while categorical variables were analyzed using the Chi-square test. Data are presented as mean ± SEM, and statistical significance was defined as *p* < 0.05.

## 5. Conclusions

This study demonstrates that JQGW improves uterine function in a letrozole-induced PCOS mouse model by suppressing the Akt2/NF-κB inflammatory pathway, modulating gut microbiota (enriching *Oscillospira*), and reversing aberrant serum metabolite profiles. These findings provide a novel mechanistic basis for JQGW in treating PCOS-related uterine dysfunction.

Future studies should validate causality using fecal microbiota transplantation and fertility-based outcomes (e.g., implantation and pregnancy rates) and explore the clinical translation of *Oscillospira* as a potential probiotic for PCOS management. The metabolomics findings presented in this study are exploratory and should be interpreted with caution, as none of the candidate metabolites passed FDR correction. Despite these limitations, the data still provide valuable exploratory insights and may guide future hypothesis-driven research, though all findings require validation in larger cohorts.

## Figures and Tables

**Table 1:** Putatively identified serum metabolites detected in the three groups.

Index	Compounds	PubChem CID	MSI Level	q Value (FDR-Adjusted *p*)
MW0057740	1,2-Dihexanoyl-sn-glycero-3-phosphocholine	10072611	3	0.713
MEDL02589	Xanthotoxol geranyl ether	5317564	3	0.713
MEDP1072	7-Methylguanosine	135445750	3	0.713
MW0104152	N-Carbamoylputrescine	502	3	0.713
MW0143708	delta-Valerolactone	10953	3	0.713
MW0156821	Ser-Leu-Leu-Ser-Phe	-	3	0.713
MW0108240	Methoxyacetic acid	12251	3	0.713
MW0063581	Sn-glycerol-1-phosphate	439276	3	0.713
MW0168667	8,11-Eicosadiynoic acid	1899	3	0.713
MW0055876	PA(20:0/16:1(9Z))	52929105	3	0.713
MW0060305	PE-NMe2(18:0/16:1(9Z))	131821098	3	0.636

Note: MSI Level 3 indicates putatively characterized compound class based on physicochemical properties or spectral similarity to database entries, without reference standard confirmation or spectral library matching.

**Table 2:** The primers for qRT-PCR.

Gene Name	FORWARD Sequence (5′-3′)	REVERSE Sequence (5′-3′)	Product Size
***Nr2f2***	TGTGCTTTGGAAGAGTACGTTA	CCAAACGGACGAAAAACAATTG	133
***Pc6***	CCAGATCTGATGCAAAACTACG	TCATTGCTTGCATCATAACGAG	92
***Ptch***	GACTTCAGGATGCATTTGACAG	GTAGAAAGCGCTCGGATTAATG	200
***Hoxa*** ***10***	CCCCTTCAGAAAACAGTAAAGC	GCTCTCGAGTAAGGTACATGTT	180
***Hoxa*** ***11***	GCTGGAGCGAGAGTTCTTCTTCAG	GACTTGACGGTCGGTGAGGTTG	91
***Hbegf***	CAAGCAAAGAAAGGAATGGGAA	GATTCTCCACTGGTAGAGTCAG	201
***Ccl5***	GACACCACTCCCTGCTGCTTTG	CTCTGGGTTGGCACACACTTGG	147
***Csf2***	GCATTGTGGTCTACAGCCTCTCAG	GGCATGTCATCCAGGAGGTTCAG	112
***Il1b***	CACTACAGGCTCCGAGATGAACAAC	TGTCGTTGCTTGGTTCTCCTTGTAC	145
***Il8***	TCGGGAGACCTCTAGACACTTTGC	GCCTGTCAAGCTGACTTCACTGG	88
***Mcp1***	CACTCACCTGCTGCTACTCATTCAC	CTTCTTTGGGACACCTGCTGCTG	96
***Mmp2***	ACCATGCGGAAGCCAAGATGTG	AGGGTCCAGGTCAGGTGTGTAAC	126
***Gapdh***	GGTTGTCTCCTGCGACTTCA	TGGTCCAGGGTTTCTTACTCC	183

Note: *Nr2f2*, nuclear receptor subfamily 2, group F, member 2; *Pc6*, proprotein convertase 5/6; *Ptch*, patched; *Hoxa10*, homeobox A10; *Hoxa11*, homeobox A11; *Hbegf*, heparin-binding EGF-like growth factor; *Ccl5*, C-C motif chemokine ligand 5; *Csf2*, colony-stimulating factor 2; *Il1b*, interleukin-1 beta; *Il8*, interleukin-8; *Mcp1*, monocyte chemoattractant protein-1; *Mmp2*, matrix metallopeptidase 2; *Gapdh*, Glyceraldehyde-3-phosphate dehydrogenase.

## Data Availability

Data is contained within the article and the Supplementary Materials.

## Supplementary Materials

The following supporting information can be downloaded at: https://www.mdpi.com/article/10.3390/ph19081153/s1, Figure S1: Representative mass spectra of chemical constituents from TCM extract; Figure S2: Serum testosterone (T) concentrations in mice; Figure S3: A Venn diagram illustrating the number of bacteria in the different groups; and Figure S4: Body weight in mice of each group. Table S1: Chemical composition of JQGW.

## Abbreviations

The following abbreviations are used in this manuscript:

AUC	area under the curve
*Ccl5*	C-C motif chemokine ligand 5
CF	cystic follicle
*Csf2*	colony-stimulating factor 2
DHT	dihydrotestosterone
E2	17β-estradiol
FINS	fasting insulin
*Hbegf*	heparin-binding EGF-like growth factor
H&E	hematoxylin and eosin
*Hoxa*11	homeobox A11
*Il1b*	interleukin-1 beta
JQGW	Jiawei Qi Gong Wan
LDA	linear discriminant analysis
*Mcp1*	monocyte chemoattractant protein-1
*Mmp2*	matrix metallopeptidase 2
NMDS	non-metric multidimensional scaling
*Nr2f2*	nuclear receptor subfamily 2, group F, member 2
PCoA	principal coordinates analysis
PCOS	polycystic ovary syndrome
*Pc6*	proprotein convertase 5/6
*Ptch*	patched
*Hoxa*10	homeobox A10
qRT-PCR	quantitative real-time PCR
SCFAs	short-chain fatty acids
TCM	Traditional Chinese Medicine
CON	control group
LET	letrozole-induced model group
JQGW-L	low-dose JQGW treatment group
JQGW-H	high-dose JQGW treatment group
MET	metformin treatment group
