# Supplementary material for: Jiawei Qi Gong Wan Improves Endometrial Receptivity in PCOS-like Mice by Attenuating Inflammation: A Multi-Omics Study Linking Gut Microbiota and Metabolite Profiles
Source: Pharmaceuticals (Basel). 2026 Jul 24;19(8):1153. doi: 10.3390/ph19081153 (PMC13516445; doi:10.3390/ph19081153)
Supplement: Supplementary file 1 [file pharmaceuticals-19-01153-s001.zip › pharmaceuticals-4273132-supplementary.pdf]

Supplementary Materials

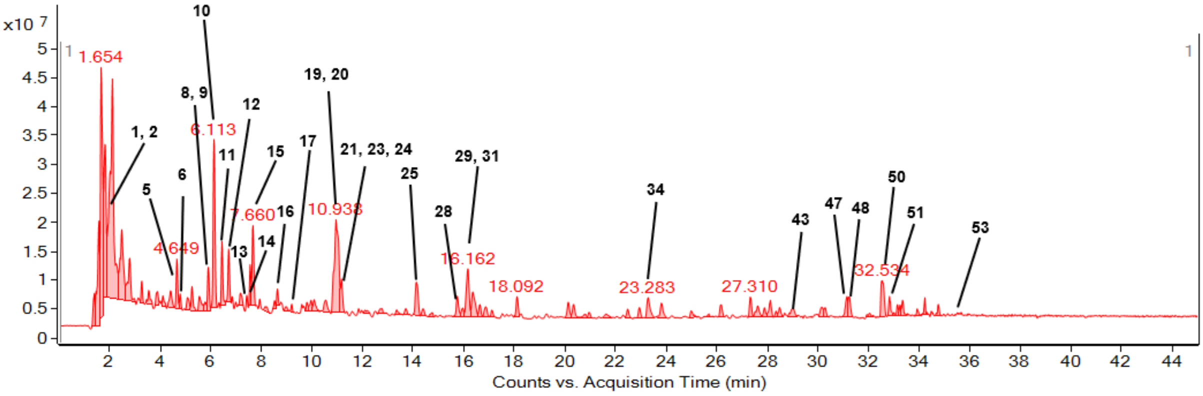

A. Total ion flow map (negative mode)

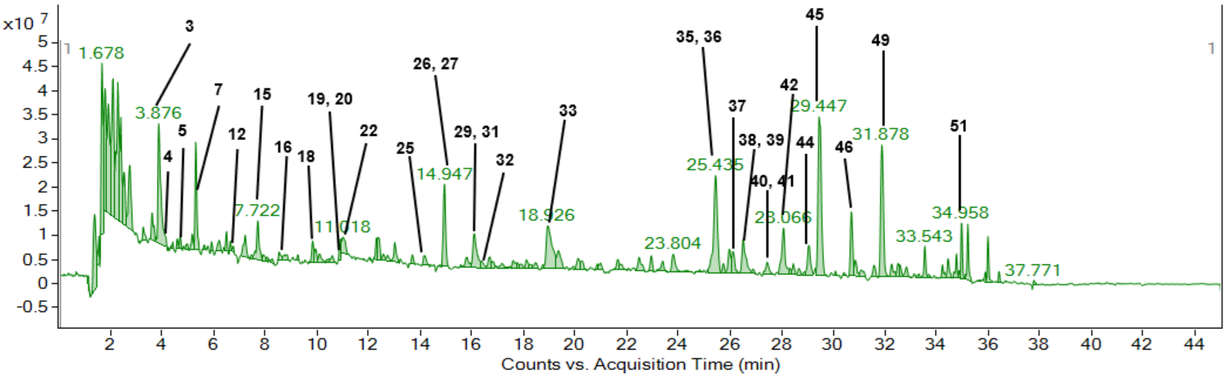

B. Total ion flow pattern (positive mode)

Supplementary Figure S1. Representative mass spectra of chemical constituents from TCM extract.

Supplementary Table S1. Chemical composition of JQGW.

Peak No.	Compound	Formula	m/z	Mass	Rt (min)	Diff(ppm)	CE	MS/MS	Species
1	citric acid	C6H8O7	191.0202	192.0275	2.750	2.69	20	87.0092, 111.0089	$[M-H]^-$
2	Gallic acid	C7H6O5	169.0147	170.0219	2.991	2.34	20	125.0246, 79.0193	$[M-H]^-$
3	phenylalanine	C9H11NO2	166.0866	165.0795	3.882	2.41	20	120.0814, 103.0545	[M+H]
4	Lycorine	C16H17NO4	288.1237	287.1165	4.082	2.61	20	178.0867, 111.0445	$[M+H]^+$
5	Neochlorogenic acid	C16H18O9	355.1030	354.0958	4.715	1.96	40	163.0390, 135.0440	$[M+H]^+$
6	Protocatechuic acid	C7H6O4	153.0199	154.0255	4.782	3.63	40	65.0038, 108.0217	$[M-H]^-$
7	tryptophane	C11H12N2O2	205.0977	204.0904	5.314	2.77	20	146.0606, 118.0656	$[M+H]^+$
8	Atractyloside A	C21H36O10	507.2475	448.2315	5.880	1.49	20	283.0492, 323.0966	$[M+HCOO]^-$
9	4-hydroxybenzoic acid	C7H6O3	137.0248	138.031	5.897	2.49	80	41.0041, 65.0040	$[M-H]^-$
10	p-hydroxylphenylpropanol	C9H10O3	165.056	166.0644	6.113	4	10	93.0348, 121.0659	$[M-H]^+$
11	Chlorogenic acid	C16H18O9	353.0888	354.0961	6.496	2.64	50	191.0564, 85.0300	$[M-H]^-$
12	cryptochlorgenic acid	C16H18O9	355.1029	354.096	6.763	1.9	20	145.0284, 163.0338	$[M+H]^+$

13	1,3-O-Dicaffeoylquinic acid	C25H24O12	515.1203	516.1268	7.413	1.44	40	191.0569, 135.0452	$[M-H]^-$
14	Caffeic acid	C9H8O4	179.0361	180.0433	7.544	5.81	20	123.0454, 152.0110	$[M-H]^-$
15	Vanillic acid	C8H8O4	167.0352	168.0424	7.660	3.99	20	105.0335, 79.0546	$[M-H]^-$
16	Vicenin II	C27H30O15	595.1668	594.1594	8.694	1.48	20	325.0760, 379.0816	$[M+H]^+$
17	Tectorigenin 4'-glucosyl (1→6) glucoside	C28H32O16	623.1623	624.1693	9.191	0.55	40	383.0762, 312.0876	$[M-H]^-$
18	N-methylphenylalanine	C10H13NO2	180.1023	179.0951	9.842	2.47	20	162.0918, 138.0916	$[M+H]^+$
19	Ferulic acid	C10H10O4	195.0657	194.0584	10.908	2.77	10	145.0285, 177.0550	$[M+H]^+$
20	liquiritin	C21H22O9	417.1200	418.1273	10.938	2.18	10	119.0506, 135.0091	$[M-H]^-$
21	Liquirtigenin-7-O-β-D-apiofuranosyl-4'-O-β-D-glucopyranoside	C26H30O13	549.1618	550.169	10.988	0.63	50	255.0661, 135.0085	$[M-H]^-$
22	Hydrangeic acid	C15H12O4	257.0814	256.0741	11.024	2.13	40	137.0234, 81.0338	$[M+H]^+$
23	neoliquiritin	C21H22O9	417.1197	418.1269	11.038	1.34	40	135.0091, 255.0665	$[M-H]^-$
24	liquiritinapioside	C26H30O13	549.1616	550.1688	11.154	0.36	40	255.0673, 135.0091	$[M-H]^-$
25	Hesperidin	C28H34O15	609.1831	610.1898	14.116	0.75	40	164.0117, 301.0719	$[M-H]^-$
26	p-methoxycinnamic acid ethyl ether	C12H14O3	207.1021	206.0943	14.937	2.42	20	189.0912, 119.0857	$[M+H]^+$

27	Senkyunolide I	C12H16O4	247.0947	224.1055	14.939	2.71	20	119.0857, 161.0962	$[M+Na]^+$
28	Isoliquiritin apioside	C26H30O13	549.1617	550.1687	15.738	0.09	40	255.0672, 135.0090	$[M-H]^-$
29	Formononetin glucoside	C22H22O9	429.1189	430.1273	16.079	1.54	20	267.0661, 252.0429	$[M-H]^-$
30	Senkyunolide H	C12H16O4	247.0946	224.1054	16.082	2.49	20	105.0699, 133.0653	$[M+Na]^+$
31	Isoliquiritin	C21H22O9	417.1201	418.1273	16.162	2.03	40	254.0585, 1148.0168	$[M-H]^-$
32	lucidumoside A	C25H34O12	527.2109	526.205	16.668	-3	40	317.0844, 395.1677	$[M+H]^+$
33	4(14),7(11)-Eudesmadien-8-one	C15H22O	219.1750	218.1677	18.932	0.49	20	203.1434, 119.0856	$[M+H]^+$
34	liquirtigenin	C15H12O4	255.0670	256.0742	23.275	2.63	20	119.0504, 135.0089	$[M-H]^-$
35	Senkyunolide A	C12H16O2	193.1229	192.115	25.442	2.31	20	90.0544, 137.0599	$[M+H]^+$
36	Isosinensetin	C20H20O7	373.1290	372.1209	25.523	2.09	50	327.0502, 153.0181	$[M+H]^+$
37	baimuxinal	C15H24O2	237.1856	236.1776	26.124	2.74	20	219.1745, 161.1324	$[M+H]^+$
38	Sinensetin	C20H20O7	373.1290	372.1209	26.507	2.26	40	343.0817, 153.0181	$[M+H]^+$
39	Shikonofuran C	C21H26O5	359.1839	358.178	26.624	-4.25	20	299.1619, 173.1323	$[M+H]^+$
40	Licoricesaponine A3	C48H72O21	985.4625	984.4551	27.606	-1.56	40	821.3937, 645.3621	$[M+H]^+$

41	licoricesaponin E2	C42H60O16	879.3981	820.388	27.857	-0.21	40	643.3465, 351.0570	[M+CH3COO]$^{-}$
42	4’,5,7,8-Tetramethoxyflavone	C19H18O6	343.1188	342.1103	28.072	3.07	50	181.0133, 153.0185	[M+H]$^{+}$
43	Uralsaponin M	C44H64O18	879.4022	880.4092	28.988	--0.05	40	643.3489, 351.0570	[M-H]$^{-}$
44	5,7,3’,4’-Tetramethoxyflavone	C19H18O6	343.1185	342.1111	29.054	2.36	20	328.0944, 282.0891	[M+H]$^{+}$
45	Nobiletin	C21H22O8	403.1395	402.1315	29.473	1.81	40	373.0927, 211.0245	[M+H]$^{+}$
46	Rehmapicroside	C16H26O8	347.1712	346.1638	30.702	2.95	20	309.1307, 225.0736	[M+H]$^{+}$
47	Licoricesaponine G2	C42H62O17	837.3915	838.3986	31.12	-0.01	40	469.3307, 141.0175	[M-H]$^{-}$
48	glycycoumarin	C21H20O6	367.1194	367.1267	31.240	1.87	40	309.0408, 201.0194	[M-H]$^{-}$
49	Tangeritin	C20H20O7	373.1289	372.1216	31.884	1.85	60	183.0299, 127.0395	[M+H]$^{+}$
50	Glycyrrhzic acid	C42H62O16	821.3970	822.4041	32.534	0.35	60	351.0572, 193.0357	[M-H]$^{-}$
51	Licoricesaponine H2	C42H62O16	821.3969	822.4038	32.817	-0.02	40	351.0573, 645.3632	[M-H]$^{-}$
52	Senkyunolide M	C16H22O4	301.1419	278.1527	34.964	3.13	20	134.0951, 201.0911	[M+Na]$^{+}$
53	Poricoic acid A	C31H46O5	497.3270	498.3341	35.978	-0.87	20	277.2183, 423.2909	[M-H]$^{-}$

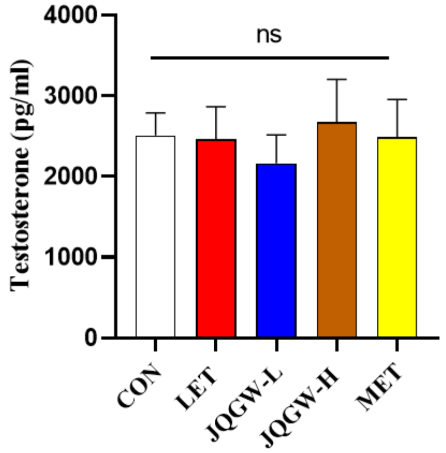

Supplementary Figure S2. Serum testosterone (T) concentrations in mice.

No significant differences in T concentrations were observed between any of the groups ($P > 0.05$).

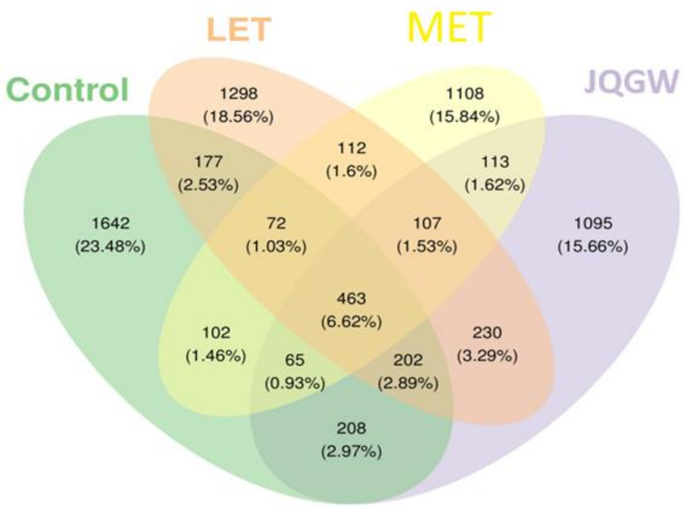

Supplementary Figure S3. A Venn diagram illustrating the number of bacteria in the different groups.

A total of 6,994 amplicon sequence variants (ASVs) or operational taxonomic units were identified across all samples. The overlap between the CON and LET groups accounted for only 13.07% of all species, and the overlap between the CON and JQGW groups was relatively higher at 13.41%, suggesting that the microbial profile in the JQGW group more closely resembled that of the CON group.

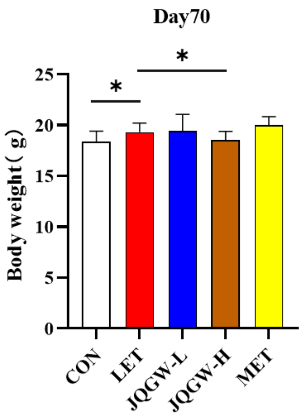

Supplementary Figure S4. Body weight in mice of each group.

Compared with the control group, the body weight in the LET group was significantly increased and could be reversed with JQGW-H treatment.
